# Point of care ultrasound - the noninvasive evaluation of hemodynamic status

**DOI:** 10.1186/2197-425X-3-S1-A543

**Published:** 2015-10-01

**Authors:** T Zawada, A Wieczorek, P Garba

**Affiliations:** 4 WSK z Poliklinika we Wroclawiu, Wrocław, Poland

## Introduction

Echocardiography(ECHO) is a useful diagnostic and monitoring tool. It allows to perform repetitive hemodynamic and functional assessment over a period of minutes, hours or days in the same patients to guide applied therapy. ECHO has been performed by our medical staff for 3 years and since then we have been observing reduced number of using invasive monitoring in our ICU.

## Objectives

The aim of the study was to analyze impact on diagnosis, decision making and management based on data received from invasive monitoring vs. echocardiography.

## Methods

Data from 2 years before and 2 years after implementing a routine echocardiography to provide hemodynamic assessments have been compared and statistical analysis has been made.

## Results

**Table 1:** *Results.*

	2011-2012	2013-2014	p
Number of hospitalized patients	666	648	NS
SAPS2	52.6	53	NS
Mean age of hospitalized patients	61	62	NS
Mean time of ICU hospitalization (days)	7.1	8.0	NS
Infections rate/1000	34.3	55.6	NS
Mean time of mechanical ventilation	6.2	8.3	NS
COST OF COLLOIDS per year (euro)	6110	2755	p < 0.05
COST OF CRISTALLOIDS per year (euro)	2985	4118.5	p < 0.05
Mortality	36.5%	36%	NS

## Conclusions

Echocardiography could be a useful method to asses patients hemodynamic status and to plan fluid or inotropic therapy. In our investigation ECHO allowed to reduce usage of fluids and

did not influence on mean time of mechanical ventilation and mortality.

## Grant Acknowledgment

ICU Staff.
